# Introducing Health Science Reports

**DOI:** 10.1002/hsr2.15

**Published:** 2017-10-16

**Authors:** Alejandro Montenegro‐Montero, Charles Young

## Abstract

Wiley is proud to announce the launch of Health Science Reports. I talked to the Editor in Chief, Dr. Charles Young, about the journal, its philosophy, and its contribution to scientific communication in the health sciences.

We are pleased to announce the launch of Health Science Reports (HSR), a new Open Access, international journal, covering all aspects of clinical research, from basic and translational studies, to public health and medical education and practice.

The journal is led by Dr. Charles Young, Chief Medical Officer at Capita Healthcare Decisions and Emergency Physician at St Thomas' Hospital, London, UK, and is supported by an international editorial board.

The journal will accept articles based on objective criteria, namely, scientific and methodological soundness, without any requirement for perceived impact or significance. We think this is an editorial stand that accelerates the communication of science and provides a better service to authors and the community as a whole. In addition, the fact that Health Science Reports is an Open Access journal means that published research will be freely and readily accessible to anyone, anywhere, allowing fast access to medical and clinical research.

I asked Dr. Young a few questions about his background and the journal.

## 1 TELL US A LITTLE ABOUT YOUR BACKGROUND

I trained in emergency medicine and still work as an Emergency Physician one day each week at St Thomas' Hospital—the other side of the river Thames from the Houses of Parliament in London. For around the last 20 years, most of my time has been spent working with healthcare research and information. Some of my jobs in the past have been Executive Editor at The Lancet medical journal, Editor in Chief at the BMJ Evidence Centre, and Vice President for Global Clinical Solutions at Wiley, where I was also lucky enough to be the founding editor of another Open Access medical journal, Clinical Case Reports. While I was at Wiley, I worked closely with the Cochrane Library editorial team and I am still part of the Cochrane Editorial Board. My interest in research really started when I was a lecturer in emergency medicine at Imperial College. I didn't particularly enjoy doing research myself (I was always awful at chemistry experiments at school!), but I became passionate about understanding how a particular piece of research affected our understanding of a clinical area or question, and how that research could be used to influence the behaviour of clinicians, patients, or carers to improve health.

## 2 WHY LAUNCH THIS JOURNAL?

Most people involved in biomedical research and publishing know that despite an increasing number of health‐related journals, it can still be very hard for authors to publish high‐quality research or opinion‐based articles. Most publications evaluate submitted research based on the novelty of the findings, and reject sound science that isn't viewed as cutting edge or doesn't fit into the journal's specific scope. This model assumes that we know now what will be viewed as important in the future. However, as understanding grows over time, it is common for research and opinion which was not seen as relevant initially to become important in a specific context. At HSR, our aim is to help address both of these issues. We will publish high‐quality research and opinion which clearly addresses important contemporary questions so allowing authors a much needed publication vehicle. In addition, we will publish high‐quality research and opinion which although does not appear to address an immediate health science need, may form an important component in the scientific record whose relevance only becomes clear at a later date. As HSR is an Open Access journal, the value of this type of research will be available to anyone who needs to access it whenever they need to access it.

## 3 WHAT MAKES HSR DIFFERENT?

There are two key differences between HSR and some other biomedical publications. The first is our relentless focus on providing the highest quality service to our authors, our reviewers, and our teams of advisers. The second important difference between HSR and other Open Access journals is our willingness to publish high‐quality submissions the importance of which will only become clear in the future. Although we may have an idea about what we need to know today, it's often much harder to understand what we will need to know tomorrow!

## 4 WHO SHOULD CARE ABOUT THIS JOURNAL?

Anyone who wants to publish high‐quality research or opinion in a health‐related area! HSR has extremely high‐quality standards and a rigorous peer review process, and we are always keen to review new submissions. We also realize that HSR may not be the first journal an author chooses to submit their work to, but our aim is to ensure every author receives an efficient and courteous service.

## 5 WHAT CAN AUTHORS, REVIEWERS, AND ADVISERS EXPECT?

An extremely good service! My view is that we are here to serve the needs of our authors, while recognising the hard work and important contributions our reviewers and advisers make. I have published in many different journals through my career and often get the impression that those journals somehow feel they are doing authors a favour by publishing their submissions. In fact, the reverse is true. At HSR, we realise how fortunate we are when authors submit their work to us, we realise how much time and effort those authors will have already invested in that work, and we realise how important it is for us to manage those submissions efficiently. Clearly, we will not be able to publish every submission we receive but we should be able to make a decision quickly and communicate effectively with our authors every time. As Editor in Chief, I am extremely fortunate to have such amazing Strategic Advisory and Editorial Boards as well as a brilliant in‐house journal team, and a very significant number of highly skilled peer reviewers. Together we can offer a really unrivalled publishing experience for authors and an amazing learning environment for all our advisers and reviewers.

## 6 WHAT'S THE MANUSCRIPT TRANSFER SYSTEM?

At Wiley, we are fortunate to publish a broad range of different health science journals. All of those journals have different styles and manuscript requirements and not all of the submissions any particular journal receives will be appropriate for publication in that specific journal. When a journal receives a high‐quality submission which is not suitable for publication, the Editor gives the authors the choice of their submission being passed on to a more suitable journal. This avoids the need for resubmission and allows the authors another chance to have their work published in an important biomedical journal. This is a really important way for us to improve the service we give to authors overall and an equally important way for HSR to see submissions which may not otherwise have been possible.

## 7 WHAT WILL HAPPEN TO HSR IN THE FUTURE?

It's hard to know how HSR will develop as it is so early in its lifecycle right now. My initial focus is to emphasise the breadth of coverage, our focus on really providing a very high‐quality service, our willingness to consider work which other journals may consider as irrelevant and our ability to work with authors to help improve the quality and impact of their publications if they need us to. I am very excited to be part of the HSR team, and as a team, we are very excited about how we can drive the future of health science research.

8

We are excited about the journal's future and its opportunity to provide an important forum for the communication of research in the health sciences. Even after a very recent launch, we are getting an increasing number of direct submissions, highlighting the immediate need for Health Science Reports. As work to improve health through the publication and dissemination of important health science research, we look forward to your participation as an author, reviewer, reader, or even better—all three!

